# Long COVID quality of life and healthcare experiences in the UK: a mixed method online survey

**DOI:** 10.1007/s11136-023-03513-y

**Published:** 2023-09-22

**Authors:** Rebecca Owen, Ruth E. Ashton, Lindsay Skipper, Bethan E. Phillips, James Yates, Callum Thomas, Francesco Ferraro, Tom Bewick, Kate Haggan, Mark A. Faghy

**Affiliations:** 1 School of Human Sciences, University of Derby, Derby, UK; 2 Long COVID Physiotherapy, London, UK; 3 NIHR Nottingham Biomedical Research Centre, School of Medicine, University of Nottingham, Derby, UK; 4 Respiratory Medicine, University Hospitals of Derby and Burton NHS Foundation Trust, Derby, UK; 5 Healthy Living for Pandemic Event Protection (HL—PIVOT) Network, Chicago, Illinois USA

**Keywords:** Long COVID, Public health, Pandemic, Morbidity, Patient and public involvement and engagement (PPIE)

## Abstract

**Purpose:**

The complexity of long COVID and its diverse symptom profile contributes to unprecedented challenges for patients, clinicians, and healthcare services. The threat of long COVID remains ignored by Governments, the media and public health messaging, and patients’ experiences must be heard through understanding of the lived experience. This study aimed to understand the lived experience of those living with long COVID.

**Methods:**

An online web-based survey was designed using Patient and Public Involvement and Engagement (PPIE) to increase understanding of the lived experiences of long COVID, and was distributed through PPIE groups, social media, and word of mouth. The survey used closed and open questions relating to demographics, pre- and post-COVID-19 health quality of life, daily activities and long COVID experiences.

**Results:**

Within our sample of 132 people living with long COVID, the findings highlight that individuals are being severely impacted by their symptoms and are unable to or limited in participating in their daily activities, reducing quality of life. Long COVID places strain on relationships, the ability to live life fully and is detrimental to mental health. Varying health care experiences are described by participants, with reports of medical gaslighting and inadequate support received.

**Conclusions:**

Long COVID has a severe impact on the ability to live life fully, and strains mental health. The appropriate mechanisms and support services are needed to support those living with long COVID and manage symptoms.

**Supplementary Information:**

The online version contains supplementary material available at 10.1007/s11136-023-03513-y.

## Introduction

Long COVID is a patient made term, defined as a condition that occurs following probable or confirmed SARS-CoV-2 infection, usually 3 months from infection with symptoms lasting for at least 2 months and no alternative diagnosis [1]. Despite initial suggestions that those with COVID-19 would likely recover in a period of weeks, it is estimated that 2 million people are living with long COVID in the UK [2], with prevalence greatest amongst individuals aged 35–49 years, females, and living in low socio-economic areas [3]. Over 200 diverse symptoms have been identified, affecting cardiovascular, pulmonary, neurological and autonomic systems, and individuals often experience their own distinctive manifestation of the condition [4, 5]. Symptoms fluctuate, with periods of remission and periods of extreme, unexpected exacerbation, often associated with preceding over-exertion [6]. One survey found that 83.3% (*n* = 1005) of people with long COVID (PwLC) experience moderate-to-poor self-reported health, moderate-to-extreme problems with daily activities (62%) and moderate-to-severe pain or discomfort (49%) [7]. Furthermore, long COVID impacts individuals ability to continue with domestic chores (84.3%), leisure (84.8%), social activities (77.1%), work (74.9%), self-care (50%), childcare (35.8%) and mental health (63.7%) [8]. Long COVID also impairs functional status, with 32.3% of individuals being unable to live alone without any assistance, and 34.5% reporting moderate to functional limitations [8].

The profile, awareness and management of long COVID and the lived experience remains overlooked by governments, the media and public health messaging [9]. In addition to determining the mechanisms of long COVID, there is a demand for health care practitioners (HCPs) and patients to work together to facilitate multidisciplinary approaches within research to develop support mechanisms, incorporating the lived experience [10–12]. Medical professionals and academics often facilitate research and decide on hypotheses and outcomes in clinical areas [13] however a movement from the National Insititue of Health Research and Funding Councils in the UK recognises the importance of involving patients throughout the research process. Patient and public involvement and engagement (PPIE) should be included in all stages of healthcare design [10, 14–16] as it provides an opportunity to embed the lived experience within research, enabling those living with illnesses to identify questions and issues that matter to them [13, 17, 18]. Research into long COVID calls for those with lived experience to have a central role within shaping the research questions and study design [9, 13].

The complexity of long COVID is reflected in the absence of effective pharmacological treatments and evidence to inform practitioners on the management of long COVID, presenting an unprecedented challenge for patients and HCPs. PwLC express concern about the absence of knowledge and understanding of long COVID, and report experiencing conflicting or inconsistent guidance from HCPs [19]. Furthermore, PwLC experience debilitating fatigue amongst many other symptoms, however by creating partnerships with patients interests at the forefront, research prioritising the patient voice can still take place whilst prioritising patients’ health [13]. Accordingly, the current study aimed to capture the impact of long COVID on quality of life (QoL) and seek recommendations for healthcare services through an exploratory online questionnaire involving no prespecified hypotheses.

## Method

Following institutional ethics approval by the Human Sciences Research Ethics Committee at the University of Derby (ETH2021-4335), a web-based survey (Qualitrcs) was distributed from October 2021-January 2022 via social media (twitter and Linkedin), word-of-mouth and PPIE networks. Participants read a participant information sheet and provided informed consent before completing the survey. All responses were anonymised by participants creating a unique identification code. Participant inclusion criteria included testing positive or suspecting COVID-19, long COVID symptoms, understanding written English and being > 18 years old. Participants were excluded if they were uncertain of the survey requirements and their answers provided in the informed consent form did not meet the required criteria.

The survey consisted of 6 sections, including 65 questions in the areas of acute and long COVID lived experience. This study focuses on the 50 questions across 5 sections relevant to the lived experience of long COVID. These sections include demographics (9 questions: age, sex, ethnicity, disability, region, relationship status, employment/occupation status), pre- and post-COVID-19 health (3 questions: pre-COVID-19 quality of life and health, post-COVID-19 quality of life and health [5-point Likert Scale; very good, good, average, below average, poor, with an open text box for further information, history of auto-immune conditions), activities of daily life (ADL) (10 questions: returning to previous activities, importance of activities, barriers) and long COVID (28 questions: care experience, obstacles to care, medical gaslighting, living with long COVID, impact on daily living, and advice for healthcare professionals [HCPs]). The survey consisted of open and closed ended questions, and participants were encouraged to provide detail surrounding their response to closed ended questions. The full survey is available in online resource 1.

### PPIE

PPIE was used throughout the research process including developing the research question, and during the creation and design of the survey. The PPIE network are established partners in the long COVID research group and long COVID physiotherapy network, external from the research group. PPIE representatives assessed the survey using their lived experience to determine survey length, content, terminology and format prior to distribution. PPIE representatives supported the circulation of the survey by sharing it within their long COVID networks and will also support dissemination of the results, by sharing findings into these support groups and forums.

### Data analysis

Closed ended questions were analysed according to frequency counts. Normal distribution was assessed for statistical data using the Kolmogorov–Smirnov test of normality (IBM SPSS Statistics v27), with Likert responses treated as interval data. Wilcoxon signed-rank tests were used to analyse within groups data, with statistical significance set to *P* < 0.05. Statistical data are presented as mean ± standard deviation (SD), with confidence interval (CI; 95%). QoL Likert scale responses were labelled as very good (1), good (2), average (3), below average (4), and poor (5) in SPSS.

The analysis of open-ended questions was guided by Braun and Clarke’s thematic analysis framework [20] by RO, CT, MF and RA. Open responses were uploaded to NVivo 12 pro (Version 12.7 QSR International, Doncaster, Australia). Following familiarisation of the data, initial codes were generated within NVivo and data were organised into groups by RO and MF. Codes were analysed, and initial themes were identified by RO, MF, RA and CT. Themes were then reviewed and defined by RO, MF, RA, CT, LS, BP, JY. The aim of the thematic analysis was to provide a narrative of the patient voice and are presented with quotes in verbatim, followed by the participant identification code in brackets. Word frequency count was also analysed within NVivo. Enhancing trustworthiness was done by using a team approach using confirmation from multiple members of the research team throughout analysis and interpretation. This is also evidenced by the audit trail from raw data through to analysis and interpretation.

## Results

### Demographics

There were 132 complete responses (85.6% female), with 32.6% of participants aged 18–40 years, 65.9% aged 41–65 years, and 1.5% > 65 years. An additional 54 responses were not included in the analysis due to participants not progressing further than the demographics section. Sample size was adjusted for missing responses when calculating frequencies. There was no missing data within the open text responses. Sample size of 132 was accepted in line with saturation of open responses [21]. Of the 132 responses, 77.3% of participants were white British, 12.9% from other white backgrounds, 5.3% white Irish and 0.8% mixed white and black Caribbean, other Black, African or Caribbean background, Indian, Pakistani, Bangladeshi, or other mixed or multiple ethnic backgrounds. Within the sample, 16.7% had a pre-existing auto-immune condition. Full participant demographic information is presented in Table 1.

**Table 1:** Participant demographics including age, sex, ethnicity and geographical location

	Demographics	*N* = (%)
Age	18–40 years	*n* = 43 (32.6%)
	41–65 years	*n* = 87 (65.9%)
	65 + years	*n* = 2 (1.5%)
Sex	Female	*n* = 113 (85.6%)
	Male	*n* = 17 (12.9%)
	Transgender	*n* = 1 (0.8%)
	Gender variant/non-conforming	*n* = 1 (0.8%)
Ethnicity	White British	*n* = 102 (77.3%)
	White Irish	*n* = 7 (5.3%)
	Other White background	*n* = 17 (12.9%)
	White and black Caribbean	*n* = 1 (0.8%)
	Other mixed or multiple ethnic background	*n* = 1 (0.8%)
	Indian	*n* = 1 (0.8%)
	Pakistani	*n* = 1 (0.8%)
	Bangladeshi	*n* = 1 (0.8%)
	Other black, African or Caribbean background	*n* = 1 (0.8%)
Geographical location	Scotland	*n* = 13 (9.8%)
	Northern Ireland	*n* = 2 (1.5%)
	Wales	*n* = 5 (3.8%)
	Northeast England	*n* = 3 (2.3%)
	Northwest England	*n* = 11 (8.3%)
	Yorkshire and Humber	*n* = 11 (8.3%)
	West midlands	*n* = 5 (3.8%)
	East midlands	*n* = 28 (21.2%)
	Southwest England	*n* = 12 (9.1%)
	Southeast England	*n* = 17 (12.9%)
	East of England	*n* = 3 (2.3%)
	Greater London	*n* = 15 (11.4%)
	Missing responses	*n* = 7 (5.3%)

Within the sample, 59.1% of participants tested positive for COVID-19, and 40.2% did not, but had symptoms consistent with COVID-19. Median time from acute infection to completion of the survey was 11.3 months, month of positive infection December 2020, and completion of the survey November 2021. During the acute COVID-19 infection phase, 87.9% recovered in community settings, 9% were admitted to hospital (4.5% < 7 days and 4.5% > 7 days), and 3% did not respond to this question. A further 3.8% of those admitted to hospital were admitted to an Intensive Care Unit (ICU). Within this sample, 76.5% of participants had been diagnosed with long COVID, 17.4% had not but report suspected long COVID and 6.1% did not disclose this information.

### Word frequency count

Word frequency count and weighted percentage was analysed in NVivo for open text responses, with ‘covid’ (count 253, weighted percentage 1.25%), ‘long’ (count 239, weighted percentage 1.18), ‘work’ (count 210, weighted percentage 1.04), ‘symptoms’ (count 169, weighted percentage 0.85) and ‘fatigue’ (count 152, weighted percentage 0.75) being the most commonly used words throughout. This data was used to inform and substantiate the development of resulting themes and to further evidence the impact on QoL and functional status.

### Descriptive statistics

**Fig. 1:**
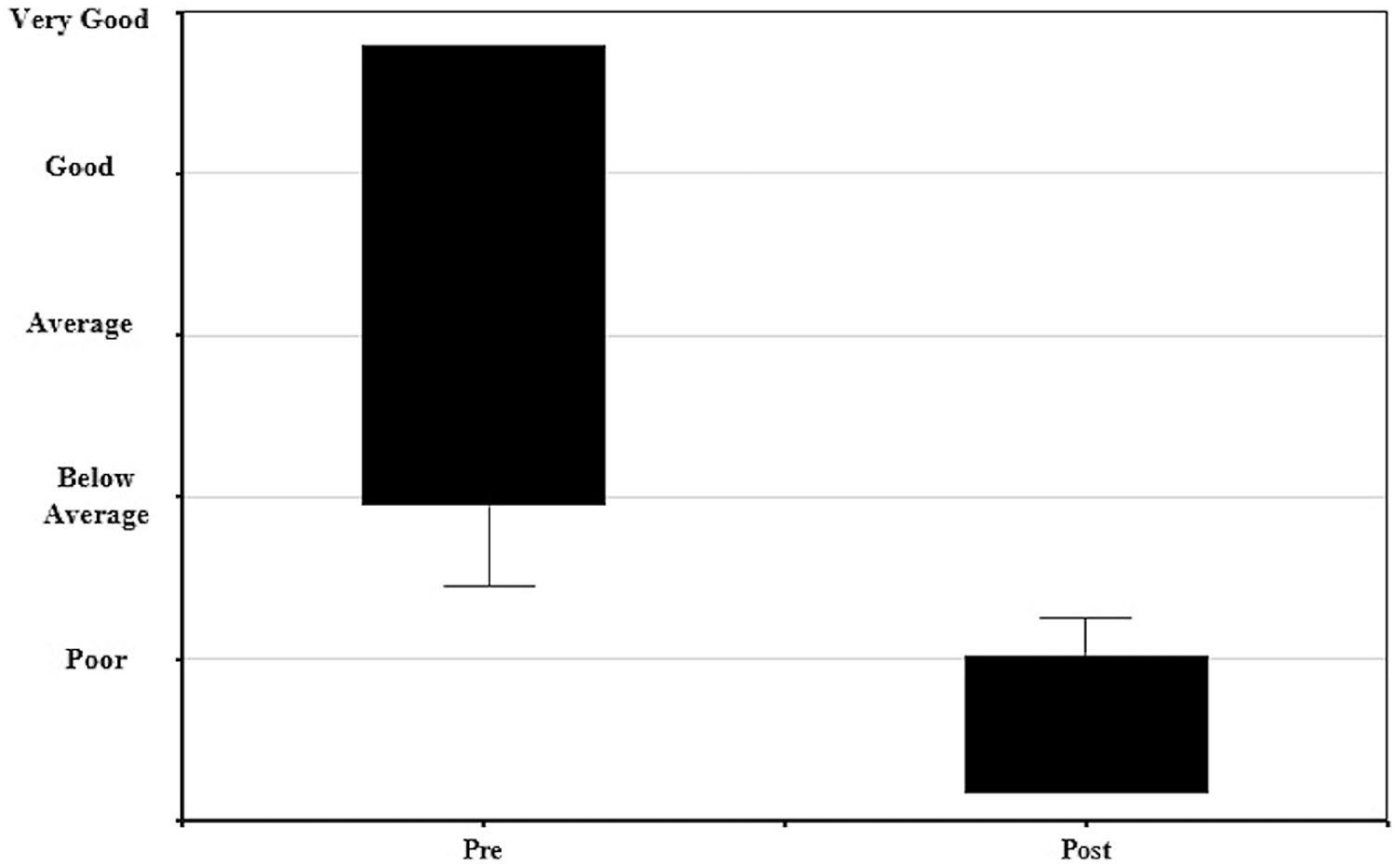
Box plot showing change in quality of life pre and post-COVID-19 infection

QoL was perceived to be higher pre-COVID-19 infection than post-COVID-19 infection (*P* < 0.01; pre-COVID-19 QoL mean 1.50 ± 0.73, 95% CI; 1.36, 1.64, post COVID-19 QoL mean 4.40 ± 0.97, 95% CI 4.23, 4.59), shown in box plot data in Fig. 1. Pre-COVID QoL and health status were reported as ‘very good’ by 52%, and 2% post-COVID-19. No participants reported ‘poor’ QoL and health status pre-COVID-19, but this was reported by 54% post-COVID-19. Furthermore, 43% (*n* = 50) were unable to return to their pre-COVID-19 activities, 38% (*n* = 44) had made a partial return to their ‘typical’ activities but symptoms still impacted their ability to engage with these activities, and 4% (*n* = 5) reported making a full return but had limitations undertaking these. Additionally, 73.5% (*n* = 97) of participants reported difficulties engaging with friends, family or colleagues and 73% (*n* = 33) of parents within this sample reported that they can no longer undertake parental responsibilities fully.

### Thematic analysis

**Fig. 2:**
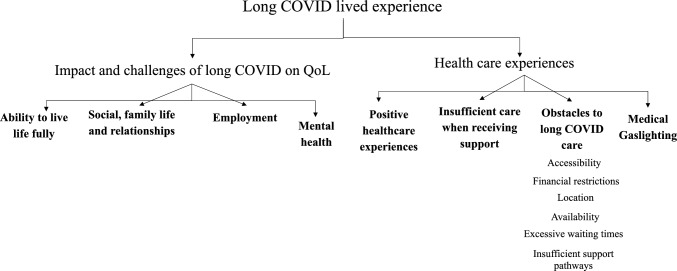
Schematic of themes

Figure 2 shows a schematic of the thematic analysis for qualitative data produced following the generation of codes and finalised themes. There were two distinct areas encompassing the lived experience of long COVID: the impact and challenges of long COVID on QoL and healthcare experiences. Further quotes to evidence themes are provided in supplementary material.

### The impact and challenges of long COVID symptoms on QoL

#### Ability to live life fully

Completely changed lifestyle, which is depressing, can’t live usual life, no energy for anything. (294EY)

Symptoms result in severe limitations of participating in daily life, with indivuduals having to change their lifestyle and sacrifice participating in their normal level of activities. When individuals do return to their typical activities, they still suffer limitations and consequences following participation. Inability to live life fully includes the ability to work, socialise, exercise and complete their previous everyday tasks.

#### Social, family life and relationships

I feel like people are fed up hearing me complain about symptoms which has made me feel isolated from friends and family. Pressure of living with reduced capabilities has impacted relationships. (294EY)

Symptoms impacting the ability to particiate in life have consequently impacted social and family life, and damaged relationships. PwLC also worry that they are burderning those around them due to changes in family roles, resulting in feelings of isolation.

#### The impact on employment

Missing work, feeling guilty about missing work. (01187DU)

PwLC who are unable to work or have reduced schedules experience feelings of guilt, financial concerns, and lack interaction with colleagues.

#### Mental health

If I didn’t have children, I’d have taken my own life a long time ago. (08126PA)

As a consequence of the impact of symptoms, PwLC experience reduced mental health with feelings of isolation, hopelessness, loss of identity and suicidal ideation.

### Long COVID health care experiences

Referral to a long COVID clinic was reported by 56% (*n* = 63) of participants, and 48% of participants had a practitioner over-seeing long COVID care (General Practitioner or long COVID clinic [*n* = 29], multidisciplinary team or specialist services (physiotherapist, immunologist, respiratory, occupational therapist [*n* = 8]). The type of care that participants received varied from commonly reported telephone appointments to a range of testing such as x-rays, blood tests, echocardiogram, and magnetic resonance imaging.

Healthcare experience themes include positive experiences, insufficient care when receiving support, obstacles to long COVID care (sub-themes; accessibility, financial restrictions, location, waiting times, availability, and insufficient support pathways), and medical gaslighting.

#### Positive healthcare experiences

2 phone calls with a (very good) OT. Provision of useful written materials, and request for GP to refer me to the local ME/CFS [myalgic encephalomyelitis/chronic fatigue syndrome] service. (056LF)

Those who describe positive healthcare experiences received mental health support, symptom management and referral to specialised routes of care. HCPs considering fatigue was also important, with 62% reporting their fatigue was taken into account, and 38% did not.

#### Insufficient care when receiving support

After a lot of struggle to access it and having been initially discharged without treatment, I have not been seen by a post-Covid clinic. (28AU)

When receiving insufficient support for long COVID care, experiences consisted of no effective interventions or treatments to support their symptoms, treatment worsening their condition such as experiencing post-exertional malaise (PEM) or post-exertional symptom exacerbation (PESE), and solely telephone calls.

#### Obstacles to long COVID care

Obstacles to accessing and receiving long COVID care were reported by 72.7% (*n* = 96) of participants. Participants reported accessibility, financial restrictions, location, excessive waiting times, availablity and insufficient support pathways as obstacles to receiving long COVID care.

#### Accessibility

My husband has to take me to most appointments because I can’t walk far. (1007)

The severe impact of symptoms on functional status such as fatigue, breathlessness and cognitive dysfunction, impact PwLCs ability to access support, such as getting to appointments, booking appointments and advocation.

#### Financial restrictions

Too expensive and already paying to see PoTS [postural orthostatic tachycardia syndrome] consultant privately. (128HH)

Private healthcare settings may have the capacity to offer testing and support for PwLC, however PwLC reported financial restrictions as a barrier to attain this.

#### Location

Long COVID research and treatments just don’t seem to exist in Northwest England. (28AU)

It also appears that there are discrepancies between services dependent on location, with long COVID clinics available in some areas of the UK and not others.

#### Excessive waiting times

Very long delay. (275FN)

After initially seeking care, patients reported extended waiting times for appointments with their GP and long COVID clinics, as well as long waits for further referrals following this.

#### Availability

They are not available on NHS. (032EH)

Long COVID care was deemed unavailable, including a lack of services, clinicians, and appointments suggesting that testing and treatment options may exist but are not readily available.

#### Insufficient support pathways

Lack of commissioning of services. Lack of knowledge of who GP can refer to. Lack of understanding. Being completely pushed from pillar to post and getting nowhere. (03–27)

When accessing and receiving support, PwLC describe lack of medical investigation, support and treatment, referral pathways and communication between medical professionals.

#### Medical gaslighting

The neurologist told me I was lying and purposely exaggerating my reflexes, also implied I was lying about other symptoms. (O2R90)

Medical gaslighting was experienced by 46% of participants. PwLC felt dismissed, disbelieved, and not taken seriously by HCPs as well as being misdiagnosed and prescribed anti-depressants to resolve their physiological symptoms. Supplementary material further highlights the prevailing experience of medical gaslighting when receiving care for long COVID, and the lack of support following this.

#### Patient recommendations for long COVID care and support

**Fig. 3:**
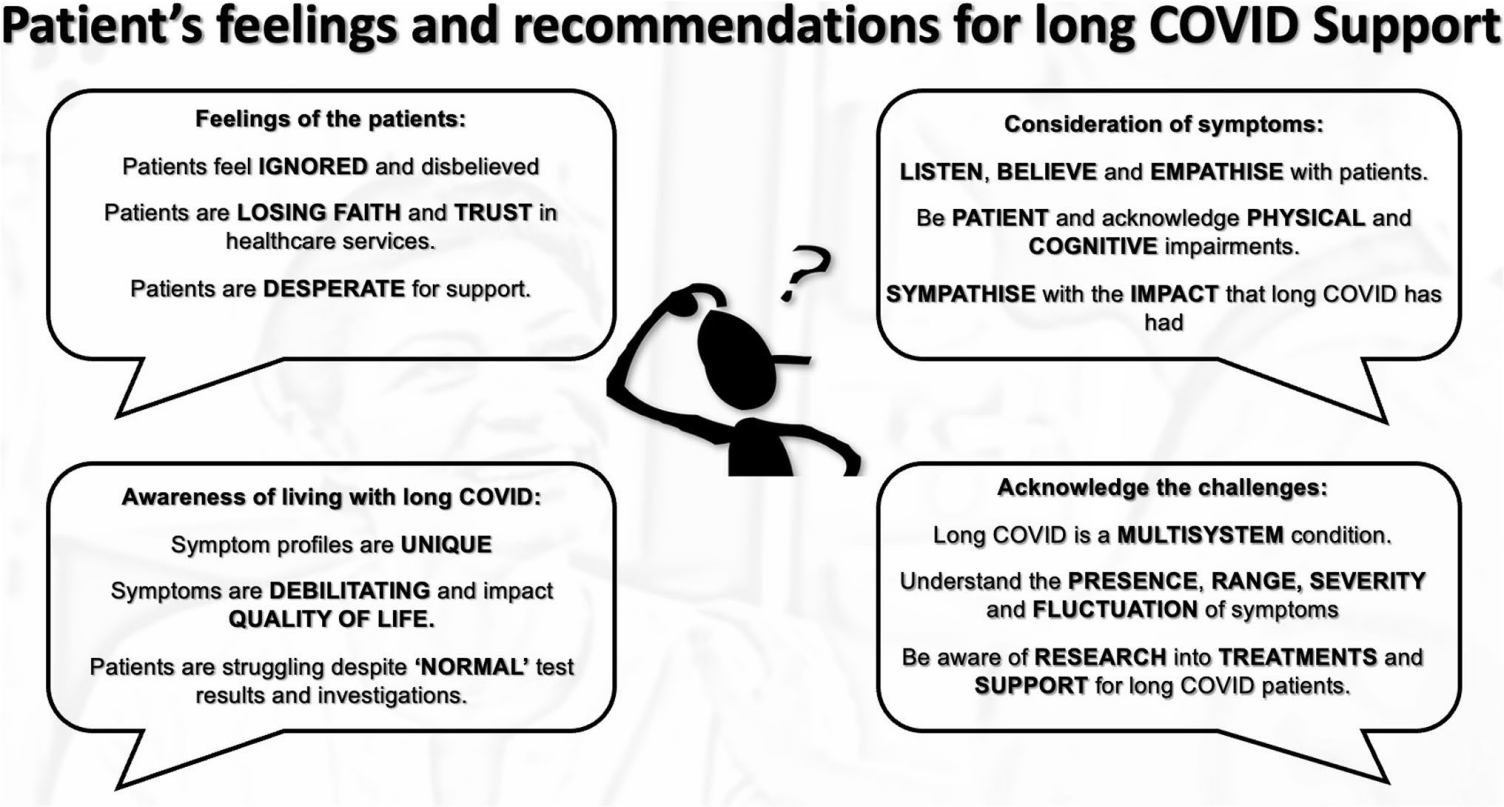
Patient recommendations for HCP helping PwLC to enhance HRQoL

As a result of the current offering of support and medical gaslighting, participants shared feedback and recommendations on how care can be improved to enhance HRQOL. These recommendations can be considered in 4 sections; communication, consideration of symptoms, awareness of living with long COVID, and the challenges of long COVID as shown in Fig. 3.

## Discussion

Our findings are consistent with previous research demonstrating that PwLC are convalescing in community settings with persistent symptoms and long-standing morbidity that primarily affects physical and mental well-being, ADL and QoL [2, 7, 8, 22]. Data here provides a deeper insight and demonstrates the broader impact that this has on social and economic determinants, that as a result further impact health and wellbeing. Here we present evidence of the adverse effect on personal and professional relationships (inclusive of relationships with healthcare professionals), an increasing reliance on friends and family for support, and psychological and emotional functioning alongside financial challenges. Evidence from previous chronic conditions has outlined the broad impacts previously, however, this is not adequately considered in conditions that are underpinned by multi-dimensional and episodic characteristics that are observed in long COVID [23–26]

The detrimental impact on mental health and wellbeing has been previously articulated and includes increased isolation, loneliness, and suicidal ideation [27]. Our data further explores the detriments and impact that inconsistencies and a lack of support and treatment received when accessing long COVID care services and the effect this has on mental and physical well-being. Specifically, patients express frustration and concern at a lack of specific and efficacious treatments and support services to eradicate and manage the condition that broadly impacts their lifestyle. Feelings of anger and frustration are possibly intensified by limited progress in the development, implementation and consistent access to efficacious support and treatments which is coupled with the manifestation and increasing reporting of isolation and loss of self-identity. The term ‘medical gaslighting’ has been widely associated with long COVID patients [28], and is a form of psychological abuse that can be intentional or unintentional and used to make victims appear or feel ‘crazy’ [29]. The term gaslighting should not be used lightly due to its critical and established use to describe both violent and non-violent abuse by an intimate partner [30]. However, medical gaslighting is an established concept with consideration to power structures within medicine separated by age, gender, social class, and race [30]. Medical gaslighting has been used by HCPs most commonly to dismiss, invalidate and provide inadequate healthcare for women’s health concerns due to the century-old stereotype that women are irrational [30]. As females are more likely to develop long COVID [31], it should not be a surprise that medical gaslighting is commonly reported by participants here when 86% of respondents are female. Other long COVID cohorts report similar experiences where HCPs did not recognise the condition, believe it existed, refused to offer testing or referral to existing services and dismissed concerns as mental health struggles [32–35].

Chronic and disabling conditions with poor diagnostic and prognostic procedures have been known to challenge medical knowledge and approaches [36, 37], and can sometimes lead to confrontation and a disconnect between patients and HCPs [38]. With complex multi-dimensional chronic diseases when HCPs are not able to explain fully explain or resolve patient issues, patients may feel as they are not being taken seriously or believed due to perceived scepticism [39–41]. It must be acknowledged that HCPs find it difficult to support patients with these conditions [42], and when HCPs are unable to provide a resolution to symptoms, feelings of helplessness may challenge their professional identity, resulting in victim blaming to allow the HCP to escape feelings of shame [36]. Furthermore, a lack of appropriate laboratory tests when investigating long COVID contributes to HCPs scepticism that long COVID symptoms have a physiological basis [33]. However, with the threat long COVID poses on individuals mental health and quality of life, it is vital that those living with the debilitating condition receive the appropriate support. For context, whilst long COVID shares overlap with other chronic conditions such as ME/CFS, there remains a dearth of understanding about the causal mechanisms that result in a broad and debilitating symptom profile that impacts health and well-being.

Existing research shows commonalities in the clinical features and pathophysiology of long COVID and ME [43]. Whilst the aetiology of long COVID is considered multifaceted with research ongoing, the links to the inflammatory state and dysregulated immune response of both conditions are similar [44, 45]. Data here demonstrates that participants report receiving treatment and care that was not helpful to their condition, with some even harmful causing PEM, such as advocating graded exercise and cognitive behavioural therapies. Importantly, research suggests that PEM must be carefully considered for long COVID, with rehabilitation and interventions incorporating pacing and strategies to minimise PEM [46]. Similarly, graded exercise therapy (GET) has been posed to cause harm in instances of ME [47–49], with the National Institute for Health and Care Excellence cautioning the use of GET for patients recovering from COVID-19 [50]. The appropriate interventions and support mechanisms are required to restore functional capacity and quality of life, and these should be created considering the recommendations of the patients suffering. As long COVID is a multifaceted, complex condition presenting with a range of physical, cognitive, and psychological symptoms, a multidisciplinary approach utilising pharmacological and rehabilitative approaches to restore functional status and QoL adopting physiatry is needed [51].

The burden of long COVID drastically impacts the global burden of disease, health and wellbeing, but it also significantly impacts healthcare services, which are already chronically underfunded and under-resourced [52, 53]. Alarmingly, waiting lists for routine treatments and procedures is affecting around 6 million people which is prominent in the most deprived areas of the UK (55% in low social-economic areas, compared to 36% in the least deprived areas) [54]. The COVID-19 pandemic has undoubtedly increased health inequality gaps and will continue to place significant strain on healthcare systems globally. Recent reports indicate that 125,000 > HCPs are unable to work due to long COVID [55] adding to existing issues with workforce capacity, and service delivery [56] at a time when the NHS is attempting to clear a backlog of over 6 million elective treatments [57]. To support the delivery of long COVID support, a collaborative approach is needed, to bring together medicine and clinical services alongside those parallel with disciplines such as exercise sciences, digital technologists, and engineering [51]. The lived experience is invaluable in enriching the understanding of long COVID and plays a key role within research [13, 17]. Research and the future design and development of long COVID services must engage patients as active stakeholders in co-creation approaches to ensure that the resultant approaches are enriched with the lived experience to ensure that patient needs are prioritised [9, 13].

The epistemic injustice of PwLC is evident, however further research is required to better understand the dynamics of the relationship with PwLC and HCPs. HCPs are subject to a lack of knowledge and understanding of long COVID. This may be partially responsible for the negative therapeutic relationship between PwLC and HCPs [58], however the repeated reporting and evidence of gaslighting is damaging to patients and the prospective future treatments and inverventions that could be beneficial to QoL. Therefore increasing the understanding and improving relationships between HCPs and PwLC is vital to foster collaboration for long COVID research, intervention development and implementation to restore HRQoL and functional status.

### Limitations

Whilst the survey received national responses throughout the United Kingdom, 86% of respondents were female and 95% of respondents reported their ethnicity as being white. Additionally, by using an online survey circulated through social media, it is likely that older participants may not have had the opportunity to participate. Further research is required to understand demographic differences that are representative of society. The survey consisted of 65 questions, all designed by those living with long COVID to ensure the lived experience would be heard. However participants were required to recall experiences which may have been challenging due to long COVID symptoms such as cognitive dysfunction and fatigue, potentially impacting the recall of information and data entry. The survey was developed and tested using patient representatives to ensure it was suitable for those living with long COVID, and participants were able to save the survey and complete it at a later date. Finally, within this sample, 40.2% of participants did not have a positive COVID-19 test. However, our study is in line with the World Health Organisation definition of long COVID which includes both probable and confirmed COVID-19 infection [1], and due to the issues regarding accuracy, accessibility and affordability of testing [14], those without a positive test have not been excluded.

## Conclusions

The lived experience of long COVID indicates that individuals are living with a severe reduction in physical and mental well-being which broadly impacts their QoL and ADL. In response to the challenges highlighted in this study, it is clear that existing support mechanisms are ineffective, sporadic, and disproportionate and there is a clear need for bespoke services that address the complex and multifaceted nature of the disease.

### Supplementary Information

Below is the link to the electronic supplementary material.

Supplementary file1 (DOCX 32 KB)

Supplementary file2 (DOCX 27 KB)

## Data Availability

Anonymised data can be made available upon request.
