# Supplementary material for: Long COVID quality of life and healthcare experiences in the UK: a mixed method online survey
Source: Qual Life Res. 2023 Sep 22;33(1):133–43. doi: 10.1007/s11136-023-03513-y (PMC10784347; doi:10.1007/s11136-023-03513-y)
Supplement: Supplementary file 1 — Supplementary file1 (DOCX 32 KB) [file 11136_2023_3513_MOESM1_ESM.docx]

Living with COVID-19, please hear what we have to say?

Unique ID Code
Unique Identification Code
 
Please use the 2 digits reflecting the day of the month you were born (01-31) followed by the last 3 characters of your postcode. This will create a 5-character unique identification code allowing us to access your data whilst keeping you anonymous.

________________________________________________________________

Display This Question:

If Living with COVID-19, please hear what we have to say.   The aim of this study is to capture and... = I have read and understood the participant information sheet

Informed consent Statement of Consent: 


1) I agree to partake as a participant in the above study.


2) I understand from the participant information sheet, which I have read in full, that this study will involve me completing one questionnaire.


3) It has also been explained to me that the risks of participation are minimal.


4) I am aware that I can withdraw my consent to participate in the procedure at any time up to two weeks after participation and for any reason, without having to explain my withdrawal and that my personal data will be destroyed and that my medical care or legal rights will not be affected.


5) I understand that any personal information regarding me, gained through my participation in this study, will be treated as confidential and only handled by individuals relevant to the performance of the study and the storing of information thereafter. Where information concerning myself appears within published material, my identity will be kept anonymous.


6) I understand that my data will be held for a maximum duration of 7 years from the commencement of the study and will be destroyed by the following date: January 6th, 2027.


7) I understand that the information collected about me will be used to support other research in the future and may be shared anonymously with other researchers. 


Please remember that you have the right to withdraw your participation. To withdraw from the study following participation, please contact m.faghy@derby.ac.uk or r.owen@derby.ac.uk providing your unique ID code.

- I consent to participating in this study and I am happy to be contacted regarding participation in future research. Please provide your email address in the text box below. (1) __________________________________________________
- I consent to participating in this study but do not want to be contacted regarding participation in future research (2)
- I do not consent to participate in this study (3)

Skip To: End of Survey If Participant Statement of Consent to Participate in the Investigation Entitled:Living with COVID-19, = I do not consent to participate in this study

End of Block: Informed consent

Start of Block: Section 1: Demographics

| Page Break |  |
| --- | --- |

Q1  Age

- 18-40 (1)
- 41-65 (2)
- 65+ (3)

Q2 To which gender identity do you most identify?

- Female (1)
- Male (2)
- Transgender female (3)
- Transgender male (4)
- Gender variant/non-conforming (5)
- Not listed (6) __________________________________________________
- Prefer not to say (7)

Q3 Please select your ethnicity/nationality:

- White British (1)
- White Irish (2)
- White Gypsy or Irish Traveller (3)
- Any other White background (4)
- White and Black Carribbean (5)
- White and Black African (6)
- White and Asian (7)
- Any other Mixed or Multiple Ethnic background (8)
- Indian (9)
- Pakistani (10)
- Bangladeshi (11)
- Chinese (12)
- Any other Asian background (13)
- African (14)
- Caribbean (15)
- Any other Black, African or Caribbean background (16)
- Arab (17)
- Any other ethnic group (18) __________________________________________________

Q4 Are you registered as having a disability?

- Yes, if so was this pre-existing or since COVID-19 infection? (1) __________________________________________________
- No (2)
- Prefer not to say (3)

Q5 Which region of the UK do you live in?

- Scotland (1)
- Northern Ireland (2)
- Wales (3)
- North East (4)
- North West (5)
- Yorkshire and Humber (6)
- West Midlands (7)
- East Midlands (8)
- South West (9)
- South East (10)
- East of England (11)
- Greater London (12)

Q83 Which region of the UK do you live in?

- Scotland (1)
- Northern Ireland (2)
- Wales (3)
- North East (4)
- North West (5)
- Yorkshire and Humber (6)
- West Midlands (7)
- East Midlands (8)
- South West (9)
- South East (10)
- East of England (11)
- Greater London (12)

Q6 What is your current relationship status?

- Single (1)
- Living with partner (2)
- Married (3)
- Separated (4)
- Divorced (5)
- Widowed (6)

Q7 Which of these best describes your current employment status?

- Employed part time (1)
- Employed full time (2)
- Self employed (3)
- Employed but currently off sick due to COVID-19 related symptoms (7)
- Employed but currently off sick for other reasons (8)
- Unemployed (4)
- Retired (5)

Q8 What is your current occupation? If this has changed since COVID-19 please detail

________________________________________________________________

End of Block: Section 1: Demographics

Start of Block: Section 2: Your COVID Diagnosis

Q1
Have you tested positive for COVID-19?

- Yes (Please indicate when in the text box provided) (1) __________________________________________________
- No, but I had symptoms consistent with COVID-19 (2)

Q2 What was your COVID-19 status:

- Recovered in a community setting (1)
- Admitted to hospital for less than a week (2)
- Admitted to hospital for more than a week (3)

Q3 If you were admitted to hospital, were you admitted to ICU?

- No (1)
- Yes, if so for how long (2) __________________________________________________

Q4 Has a clinician diagnosed you with Long COVID?

- Yes, if so which clinician and when was this (date) (1) __________________________________________________
- If you haven't had a diagnosis but you suspect you have Long COVID, please select here (2)
- Prefer not to say (4)

End of Block: Section 2: Your COVID Diagnosis

Start of Block: Section 3: General Questions about your pre COVID-19 and post COVID-19 state

Q1 How would you describe your quality of life and general health before you were diagnosed or believe you contracted COVID-19? Please feel free to tell us why you selected this answer

- Very good (1) __________________________________________________
- Good (2) __________________________________________________
- Average (3) __________________________________________________
- Below average (4) __________________________________________________
- Poor (5) __________________________________________________

Q2 How would you describe your quality of life and general health since you were diagnosed or believe you contracted COVID-19? Please feel free to tell us why you selected this answer

- Very good (1) __________________________________________________
- Good (2) __________________________________________________
- Average (3) __________________________________________________
- Below average (4) __________________________________________________
- Poor (5) __________________________________________________

Q3 3. Do you have a history of other auto-immune issues prior to being diagnosed or believe you contracted COVID-19? Please feel free to tell us why you selected this answer

- Yes (1) __________________________________________________
- No (2) __________________________________________________
- Prefer not to say (3) __________________________________________________

End of Block: Section 3: General Questions about your pre COVID-19 and post COVID-19 state

Start of Block: Section 4: Acute COVID-19

Q1 Did you get a test for COVID-19 in the first few days of symptoms?

- Yes (1)
- No (2)

Display This Question:

If Did you get a test for COVID-19 in the first few days of symptoms? = No

Q1a If you did not get a test for COVID-19 in the first few days of symptoms, please tell us why not?

________________________________________________________________

Display This Question:

If Did you get a test for COVID-19 in the first few days of symptoms? = No

Q1b Do you believe that not having a test in the first few days of symptoms impacted upon you getting medical help? Please feel free to tell us why you selected this answer

- Yes (1) __________________________________________________
- No (2) __________________________________________________
- Prefer not to say (3)

Q2 Can you describe your experience during the acute phase (i.e, the first 6 weeks) of COVID-19 infection?

________________________________________________________________

Q2a Can you describe how you were managed during the acute phase, considering medical management (i.e, the first 6 weeks) of COVID-19 infection?

________________________________________________________________

Q2b Can you describe what you did to manage your symptoms during the acute phase (i.e, the first 6 weeks) of COVID-19 infection?

________________________________________________________________

Q3 Do you believe you had adequate care during the acute phase (i.e, the first 6 weeks) of COVID-19 infection?

- Yes (1)
- No (2)

Display This Question:

If Do you believe you had adequate care during the acute phase (i.e, the first 6 weeks) of COVID-19... = No

Q3a If you did not receive adequate care during the acute phase (i.e, the first 6 weeks) of COVID-19 infection, please tell us why not?

________________________________________________________________

Q4 What do you wish you had known about the acute phase to help with your recovery?

________________________________________________________________

Q5 Are there any ‘safety’ issues or developing symptoms/pathologies that people who have acute COVID-19 need to look out for?

________________________________________________________________

Q6 What symptoms should alert clinicians or patients to seek urgent medical help?

________________________________________________________________

End of Block: Section 4: Acute COVID-19

Start of Block: Section 6: Activities of daily life

Q1 Have you experienced any difficulties engaging with friends, family, or colleagues regarding your symptoms? Please feel free to tell us why you selected this answer

- Yes (1) __________________________________________________
- No (2) __________________________________________________
- Unsure (3) __________________________________________________

Q2 Since your COVID-19 infection, have you been able to return to your usual activities of daily life? This could include social and leisure activities. Please can you provide some details on this

- Yes (1) __________________________________________________
- Partially (2) __________________________________________________
- No (3) __________________________________________________

Q3 What leisure activities do you like to engage in? (i.e, hobbies, favourite past times, recreational activity)

________________________________________________________________

Display This Question:

If Since your COVID-19 infection, have you been able to return to your usual activities of daily lif... = Yes

And Since your COVID-19 infection, have you been able to return to your usual activities of daily lif... = Partially

Q4 If you have been able to return to your leisure activities, how long after your COVID-19 was this?

- In a matter of weeks (1)
- After a month or more (between 1-3 months) (2)
- After a several months (>3months) (3)
- I’ve not been able to return to these activities (4)

Q5 How important are these activities to you? We'd like to hear more about how important these activities are to you, please can you provide some details on this

- Very important to me (1) __________________________________________________
- Somewhat important to me (2) __________________________________________________
- Not at all important to me (3) __________________________________________________

Display This Question:

If Since your COVID-19 infection, have you been able to return to your usual activities of daily lif... = Yes

And Since your COVID-19 infection, have you been able to return to your usual activities of daily lif... = Partially

Q6 What support if any do you need to undertake these activities?

________________________________________________________________

Display This Question:

If Since your COVID-19 infection, have you been able to return to your usual activities of daily lif... = No

Q7 If you have not been able to return to these activities, what would you say is the biggest barrier for you?

________________________________________________________________

Q8 Are you a parent with childcare responsibilities?

- Yes (1)
- No (2)

Display This Question:

If Are you a parent with childcare responsibilities? = Yes

Q9 Do you feel you are able to undertake these responsibilities fully? We'd like to hear more about this, please can you provide some details on this.

- Yes (1) __________________________________________________
- No (2) __________________________________________________

Display This Question:

If Are you a parent with childcare responsibilities? = Yes

Q80 Are you experiencing any difficulties fulfilling your childcare responsibilities? If so, please state what these are and how they are being impacted.

- Yes, always (1) __________________________________________________
- Sometimes (2) __________________________________________________
- No, not at all (3)

End of Block: Section 6: Activities of daily life

Start of Block: Section 7: Long COVID

Q1 Do you have anyone over-seeing your long COVID care?

- Yes (1)
- No (2)

Display This Question:

If Do you have anyone over-seeing your long COVID care? = Yes

Q1a If someone is over-seeing your long COVID care, please provide details of who or where you receive this care from?

________________________________________________________________

Q2 Have you been referred to a long COVID clinic?

- Yes (1)
- No (2)

Display This Question:

If Have you been referred to a long COVID clinic? = Yes

Q2a If you have been referred to a long COVID clinic, how long did you wait to see someone after you had caught COVID-19?

________________________________________________________________

Display This Question:

If Have you been referred to a long COVID clinic? = Yes

Q2b If you have been referred to a long COVID clinic, what did your care consist of?

________________________________________________________________

Q3 If you have accessed long COVID care, have these services taken into account your fatigue when assessing you? Please give details on how they have or have not

- Yes (1) __________________________________________________
- No (2) __________________________________________________

Q4 Have you experienced any obstacles to receiving care for your long COVID?

- Yes, if so please give details (1) __________________________________________________
- No (2)

Q5 5. Have you experienced any medical gaslighting i.e. where someone has blamed your symptoms on psychological factors such as anxiety?

- Yes, if so can you describe this? (1) __________________________________________________
- No (2)

Q6 What have been your main difficulties in living with long COVID?

________________________________________________________________

| Page Break |  |
| --- | --- |

Q6a How have these impacted upon your activities of daily living?

________________________________________________________________

Q7 Have you experienced or are you receiving support for mental health issues because of COVID-19 related symptoms?

- Yes (1)
- No (2)
- Prefer not to say (3)

Display This Question:

If Have you experienced or are you receiving support for mental health issues because of COVID-19 re... = Yes

Q7a If you experienced mental health issues or are receiving support for mental health issues because of your COVID-19 experience, would you be willing to tell us more about this?

- Yes, please detail below (1) __________________________________________________
- No, I'd prefer not to (2)

Q8 Has long covid affected your cognitive abilities? (E.g. thinking, knowing, remembering, judging, and problem-solving)

- Yes, if so how? (please detail) (1) __________________________________________________
- No (2)

Q9 How do you think long COVID has affected you emotionally i.e. your ability to deal with different emotions such as stress, happiness, worry etc?

________________________________________________________________

Q10 How does exercise or activities impact upon your symptoms?

- Not at all (1)
- Very mildly (2)
- Mild (3)
- Moderately (4)
- Severely (5)

Q11 How do your exercise levels compare between now and pre-covid?

- Far below pre-covid (1)
- Below pre-covid (2)
- The same as pre-covid (3)
- Above pre-covid (4)
- Far above pre-covid (5)

Q12 What systems in your body do you feel have been affected by Long Covid?

- Skeletal (bones) (1)
- Muscular (muscle) (2)
- Nervous (brain) (3)
- Endocrine (hormonal) (4)
- Cardiovascular (heart) (5)
- Lymphatic (immune function) (6)
- Respiratory (lungs, airways) (7)
- Digestive (8)
- Urinary (9)
- Reproductive (10)
- Other (please detail) (11) __________________________________________________

Q13a What treatments have you received for long COVID that have been the most helpful?

________________________________________________________________

Q13b What lifestyle changes have you made that have been helpful in managing your long COVID?

________________________________________________________________

Q13c What treatments/ lifestyle changes do you feel would help you but you may not have access to them?

________________________________________________________________

Q13d Why can you not access the treatments/ lifestyle changes you feel would help you?

________________________________________________________________

Q14 What do health care professionals need to know when assessing and treating someone with long COVID?

________________________________________________________________

Q15 What has been positive or negative about being unwell with an unknown/novel virus?

________________________________________________________________

Q16 Have you been taking any supplements or medication for long COVID?

- Yes, if so please detail (1) __________________________________________________
- No (2)

Display This Question:

If Have you been taking any supplements or medication for long COVID? = Yes, if so please detail

Q17 Have the supplements or medication you have been taking for long COVID been helpful or made symptoms worse? Please give us more details on this

- Helpful (1) __________________________________________________
- Made symptoms worse (2) __________________________________________________
- Neither helpful nor made symptoms worse (3) __________________________________________________

Q18 Have you had any cardiac symptoms? (Heart palpitations, increase heart rate, blood pressure)

- Yes, if so how have they been dealt with? (please detail) (1) __________________________________________________
- No (2)

Q19 Have you noticed any changes in weight?

- Yes, if so have these been associated with gut symptoms? (please detail) (1) __________________________________________________
- No (2)

Q20 Have you had to change what you eat since having long COVID?

- Yes, if so why and how (1) __________________________________________________
- No (2)

End of Block: Section 7: Long COVID
