# Supplementary material for: Long COVID quality of life and healthcare experiences in the UK: a mixed method online survey
Source: Qual Life Res. 2023 Sep 22;33(1):133–43. doi: 10.1007/s11136-023-03513-y (PMC10784347; doi:10.1007/s11136-023-03513-y)
Supplement: Supplementary file 2 — Supplementary file2 (DOCX 27 KB) [file 11136_2023_3513_MOESM2_ESM.docx]

| **Themes** | **Raw Quotes** | **ID** |
| --- | --- | --- |
| **Impact and challenges on QoL** | | |
| Ability to live life fully | When I’m tired, I feel like I have early-onset dementia- extreme confusion | 10975 |
|  | I’m now mostly housebound | 276BJ |
|  | I am largely housebound by long COVID | 185QQ |
|  | I get PEM now (even after slight exertion). I need to nap a few times every day. No energy. Lots of pain - recently diagnosed fibromyalgia, still breathless & dizzy & extremely thirsty every day, still chestpains, etc.. | 10975 |
|  | I cannot walk far, I get PEM | 10-07 |
|  | For a day of work, I currently need a day of rest | 25BS8 |
|  | Not yet able to exercise, limited ADL’s and social activities | 04043LZ |
|  | Some weeks better than others, on bad weeks still hard to have enough energy to do more than caring for my kids, cooking meals for family, food shopping. It is hard to walk long distances or uphills (I live in mountains so this is unavoidable), husband away traveling for business often makes it hard to get through the days but I do as I have kids and one with long Covid | 28315 |
|  | I have returned to work because I have no choice but to work, other than that not really. I dont have the energy | 121BT |
|  | no energy to do what I want to do, always having to settle for less. Losing my ability to focus, forgetting words, and losing my train of thought. | 013UD |
|  | I dont really have a life now, all i want to do is rest and sleep | 04077WQ |
|  | I need to rest for days on end. I can't even cook. I don't clean. I still just survive | 10-07 |
|  | My life has completely changed for the worse. Im now on benefits | 039TP |
|  | totally I'm at a dead end as I can't swim can't excercise with help as there's nowhere to do it safely | 202TN |
|  | Limits what I can comfortably do | 225AW |
|  | I can do things, but it is exhausting. Sometimes I get home and get into bed. It takes me longer to recover from physical activities | 174TH |
|  | I manage to work from home but have to go to bed at 8:30 every night and barely leave the house. I am barely able to do anything other than my work | 288RS |
|  | I have spent most of the past 20 months too I’ll even to self care. Never mind live a life or care for my children | 02010JD |
| Social, family life and relationships | Not going out. Too scared | 10312QS |
|  | I have been struggling with my emotional/ physical relationship with my husband. As week as the reduced wages into the home and increase of workload for my husband. I used to care for my grandfather but unable to do so for now. | 25117PG |
|  | holding back from joining in | 028EP |
|  | reduced social participation, reduced ability to perform daily activities, reduced participation in life roles, reduced ability to plan for the future, impaired family and relationship roles | 225FD |
| Employment | Really miss doing my job which I loved | (10975) |
|  | Long term sickness from work | 150RS |
|  | I could lose my job due to the time it's taking and the time I have had to have off work | 0327 |
|  | I am back working, but I am exhausted, and every day is a struggle. But as a single person who self-employed I have no choice but to work to pay bills and put food on the table | 298JD |
| Mental health | The isolation and hopelessness | 106PS |
|  | Loneliness, trying to accept that I’m not me anymore | 233LU |
|  | Struggling to compose texts and emails and to complete simple tasks is deeply upsetting for me and results in my mental health taken a severe knock | 013UD |
| **Healthcare experiences** | | |
| Positive | Advice on how to manage symptoms/ POTS, sign posted to online advice/support. Review of my ECHO, ECG, treadmill test | 25117PG |
|  | Management of symptoms e.g., pacing | 04077EQ |
|  | Phone call and email. Support, referrals to clinics and clinicians, medication, X-rays, and they set up the Derbyshire LC Facebook group which is ACE! | 201BY |
|  | Health psychology – coming to terms with new me, the unknown for the future and mindfulness | *0330* |
|  | Place to unload | *25117PG)* |
|  | Vocational support, chronic fatigue support, breathing support group, memory support | *043FW* |
|  | Referral to an Occupational Therapist and to the fatigue clinic | *106PS* |
|  | Referral to respiratory medicine and long covid physio | *188TD* |
| Insufficient care provided | A chat about pacing, fatigue and breathing nothing else, never heard off them since | 28RAX |
|  | Very patchy care from gp practice | 25bs8 |
|  | One OT phone call, a face to face appt with a rehab doctor to confirm that it was long covid, then discharged to GP management | 185QQ |
|  | Telephone triage with signposting to self care information | 028EP |
|  | Phone calls with a junior physio (unhelpful) | 12022DL |
|  | Not a lot. Feels like a triage system. | 194JU |
|  | 2 phone calls with a clinician. Advice to look at self care strategies for POTS. Promise of referral to cardiology which didn't happen so my GP referred me instead. | 056LF |
|  | Only physically seen Respiratory Clinician, all other contact by phone or email | 018ET |
|  | Not offered any help for any of my symptoms. | 04847DD |
|  | Referred to ENO breathing course otherwise no other treatment offered | 032EH |
|  | Disappointed with level of care provided | *032EH* |
|  | Disappointing! A respiratory consultant who only sees respiratory things | *10975* |
|  | Did refer me to rehab/physio but after 3 sessions I had to stop during breathing difficulties and chest pain so was advised to stop | *128EB* |
|  | Giving me exercises that caused weeks long relapses and telling me to ask GP for anything else | *066QL)* |
| **Obstacles to long COVID care** | | |
| Accessibility | Not well enough to access | 202TN |
|  | I struggle to get care for my children. I don’t have it in me to advocate for myself | 032EH |
|  | Getting to appointments alone | 283RD |
| Financial restrictions | Nowhere will do it for a small amount of money | 013UD |
|  | I cannot afford to go private | 0330 |
|  | Financial reasons | 153RX |
|  | Not available for a reasonable price in a location I can get to | 060HP |
| Location | There is no availability locally, I have been refused referral for consultation with specialists within the NHS. | 0330 |
|  | Long COVID clinics were unavailable here in Wales | 160EW |
| Excessive waiting times | Excessive waiting list time | 118JQ |
|  | Waiting list long | 294EY |
|  | Long waiting lists for appointments | 14011TS |
|  | Long wait times | 106PS |
|  | It has taken 19 months to get any care at all | 244AZ |
|  | Long wait | 0128SHF |
| Availability | not available on NHS | 0330 |
|  | Poor availability | 07034UU |
|  | Lack of available clinicians | 251AJ |
|  | Given up asking GP every month | 02010JD |
| Insufficient support pathways | Misdiagnosed with anxiety. Lack of tests, scans & assessments available from NHS. Had to rely on private consultants to have necessary tests etc. Cardiologist, Neurologist, Rheumatologist, Lung specialist, physiotherapists, Gastroenterologist etc. Long waiting list for LC clinic. GP not taking plethora of symptoms after acute phase (many months) until I produced reports from cardiology & physiotherapist. | 032EH |
|  | Limited treatment option, very difficult to access an OT assessment, difficult to attend appointments as they trigger fatigue but majority have been done via video/ phone and now physio come to my house if face to face is necessary | 153RX |
|  | I had to beg my GP to refer me | 225FD |
|  | No treatment. No care. No support. No financial support. Fighting all the time to be believed to have serious health concerns that Long COVID mimics ruled out | 08126PA |
| Medical gaslighting | One consultant said I’ll talk to you when you’re 10 stone lighter | 21123ES |
|  | The doctor dismissed my tinnitus as being a recognised symptom of Long COVID. I felt she was dismissive and that I was neurotic. I got upset at not being taken seriously or being shown any empathy following this flare in symptoms or what the cause might be. The doctor prescribed me a course of anti-depressants | 21123ES |
|  | Disbelief of GP, disbelief of neurologist during admission for covid symptoms | 02R90 |
|  | My GP has said that my weight is causing the symptoms and I should loose weight to feel better. Told to increase exercise activity to feel better | 25117PG |
|  | have been prescribed anti-depressants to see if they work before referral to long covid clinic. One gp did not believe long covid existed | 275FN |
|  | Attended A&E on gp advice for rash and bruising and was told in July 2020 that long covid was likely to relate to mass hysteria induced by social media | 25BS8 |
|  | A doctor told me my total brainfog "couldbe menopausal"! (I'm not menopausal)Yes, a rheumatologist told me to "Get out more & exercise more" - despite my PEM & breathlessness. A cardiologist told me to "Push the boat out! | 10975 |
